# Effects of data-driven respiratory gating on visualization and quantification of breast and upper abdominal cancers in FDG PET/CT examinations

**DOI:** 10.1007/s12149-025-02017-8

**Published:** 2025-01-23

**Authors:** Mitsuaki Tatsumi, Naomi Morita, Akira Kida, Risa Momoi, Kayako Isohashi, Atsuya Okada, Noriyuki Tomiyama

**Affiliations:** 1 Department of Radiology, The University of Osaka Hospital, 2-2 Yamadaoka, Suita, Osaka 565-0871 Japan; 2 Jinsenkai MI Clinic, Toyonaka, Japan; 3 Department of Radiology, The University of Osaka Graduate School of Medicine, Suita, Japan

**Keywords:** PET, Data-driven respiratory gating, Breast, Liver, Pancreas, Abdomen

## Abstract

**Objective:**

Data-driven respiratory gating (DDG) has recently been introduced to improve image quality in the PET portion of PET/CT examinations. The latest DDG system does not require any external equipment or extended examination time. In this study, we investigated the effects of the new DDG system on the visualization and quantification of breast and upper abdominal cancers, comparing the results with those obtained using the standard free-breathing (STD) PET protocol.

**Methods:**

A total of 223 cancer lesions (138 breast and 85 upper abdominal) evaluated with FDG PET/CT were included in this study. PET images were reconstructed using the STD and DDG algorithms. Lesion blurring and conspicuity were each visually graded on a three-point scale. The longest diameter (LD), SUVmax, and metabolic tumor volume (MTV) of the lesions were used for quantitative analysis. % change in SUVmax or MTV was calculated from the metrics in STD and DDG images. Fifty-six texture features (TFs) were also evaluated. Visual scores and quantitative metrics were compared between STD and DDG images. % change in SUVmax or MTV was compared in the lesion location groups or in the high and low groups based on LD, SUVmax, or MTV in STD images.

**Results:**

Visual scores for lesion blurring and conspicuity were both significantly higher in DDG than in STD PET images. SUVmax and MTV were significantly higher and lower, respectively, in DDG than in STD images. An increase in SUVmax and a decrease in MTV were observed in 96% and 86% of all lesions, respectively. Group analysis revealed that % change in SUVmax was greater in the upper abdominal than the breast lesions and % change in MTV was greater in the high LD and high MTV groups than in the low LD and low MTV groups, respectively. Quantitative changes in TFs were observed between STD and DDG images for most of the features.

**Conclusion:**

This study demonstrated that DDG improved visualization and quantification of breast and upper abdominal cancers in FDG PET/CT examinations. DDG PET images exhibited an increase in SUVmax, a decrease in MTV, and changes in TFs.

**Supplementary Information:**

The online version contains supplementary material available at 10.1007/s12149-025-02017-8.

## Introduction

In chest and abdominal imaging, respiratory motion causes image degradation, and various attempts have been made to overcome this problem. Breath holding is a simple method, but it is not suitable for PET imaging, which requires relatively long data acquisition time. Respiratory gating methods have been used for PET imaging as alternatives [1]. However, traditional gating methods require dedicated devices, which limits their widespread adoption. Recently, device-less data-driven respiratory gating (DDG) methods have been developed and have gained attention [1–3]. The latest DDG system does not even require extended examination time. Although some reports have demonstrated the usefulness of DDG in evaluating lung cancer [3–6], there are few reports on breast and upper abdominal cancers, which are also affected by respiration.

In this study, we investigated the effects of the new DDG system on the visualization and quantification of breast and upper abdominal cancers, comparing the results with those obtained using the standard free-breathing (STD) PET acquisition protocol.

## Materials and methods

This retrospective study was approved by the Ethical Review Board of The University of Osaka Hospital (No. 22384-2), which waived the requirement for informed consent.

A total of 223 cancer lesions evaluated with FDG PET/CT between March 2022 and January 2024 were included in this study. The 223 lesions included 138 breast and 85 upper abdominal cancers. The 85 upper abdominal cancers consisted of 67 liver and 18 pancreatic cancers. Because the lower portion of the liver and pancreas are approximately the same distance from the diaphragm, which is closely associated with respiratory motion, liver and pancreatic lesions were treated together as upper abdominal lesions unless otherwise indicated. If multiple lesions were present in the target breast, liver, or pancreas, the largest lesion was included in this study.

The lesion location and longest diameter (LD) obtained from the imaging studies are summarized in Table 1.

**Table 1:** Lesion location and longest diameter (mm)

Location		Numbers				LD median (IQR)
		Total	LD < 20	20 ≦ LD < 40	40 ≦ LD	
All		223	60	122	41	25 (19.5–35.0)
	Breast	138	48	73	17	20 (15.0–30.0)
Upper abdomen		85	12	49	24	29.9 (23.3–40.7)
	Liver	67	10	34	23	32.6 (22.4–43.7)
	Pancreas	18	2	15	1	28.4 (23.8–31.9)

*LD* longest diameter, *IQR* interquartile range

### FDG PET/CT imaging

FDG PET/CT imaging was performed using a Biograph mCT scanner (Siemens Medical Solutions, Knoxville, USA). PET images were acquired with continuous bed motion with a 0.9 mm/s bed speed 60 min after intravenous injection of FDG at a dose of 3.7 MBq/kg body weight. The PET field of view was 78 cm. Images were reconstructed using OSEM (subset 21, iteration 2, and Gaussian filter 5 mm) and time-of-flight systems. All PET images were reconstructed with a 200 × 200 matrix.

In the DDG algorithm evaluated in this study (OncoFreeze AI), respiratory waveforms were derived directly from PET list-mode raw data during continuous bed motion without any external device such as the monitoring system or abdominal bandage, and then used to reconstruct PET images. The image reconstruction also used the motion blurring information estimated from mass preservation optical flow, allowing 100% utilization of the acquired PET counts without extended data acquisition time. The respiratory waveform required for DDG is measured every 500 ms and the bed moves 0.45 mm during this time. Therefore, the bed movement is not considered to affect the DDG effect. The details of the DDG algorithm used in this study have been described elsewhere [2, 6].

### Image analysis

PET images reconstructed using the STD and DDG algorithms were interpreted separately, and visual and quantitative analyses were performed.

#### Visual analysis

**Fig. 1:**
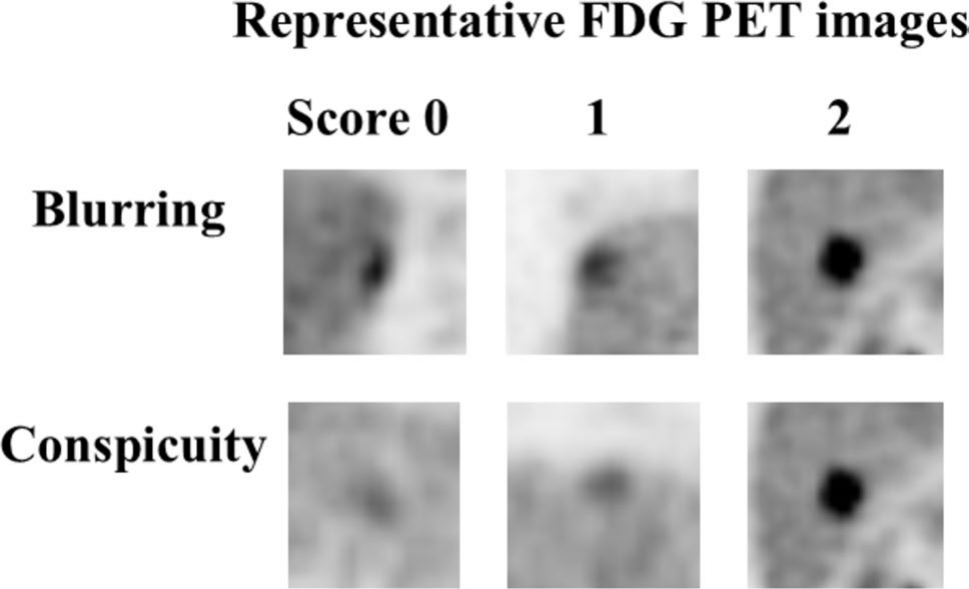
Representative FDG PET images corresponding to each score for lesion blurring and conspicuity. The status of lesion blurring and conspicuity on FDG PET images was expressed using the following scores: 0: poor, 1: good, and 2: excellent

The STD and DDG PET images were visually interpreted separately by two board-certificated nuclear medicine physicians, and the status of lesion blurring and conspicuity were each graded on a three-point scale (0: poor, 1: good, and 2: excellent). The scores were recorded, and discrepancies, if any, were resolved by consensus between the two observers. Representative FDG PET images corresponding to each score for lesion blurring and conspicuity are shown in Fig 1.

#### Quantitative analysis

LD, the maximum value of standardized uptake value (SUVmax), and metabolic tumor volume (MTV) of the lesions were used for quantitative analysis. SUVmax was calculated from a single voxel showing the maximum SUV in each lesion. MTV was defined as the volume within the tumor margin delineated as 40% of SUVmax. These quantitative metrics were obtained using commercially available software (PETSTAT: AdIn Research, Tokyo, Japan). The percentage of change (% change) in SUVmax or % change in MTV was defined as [(metrics in DDG/metrics in STD - 1) × 100] (%).

Texture analysis was performed as a part of the quantitative analysis. Fifty-six texture features (TFs) were evaluated in this study, and entropy and homogeneity calculated from the co-occurrence matrix, low gray-level zone emphasis (LGZE) and high gray-level zone emphasis (HGZE) from the gray-level zone length matrix, and short-run emphasis and long-run emphasis from the gray-level run length matrix were included as recommended in the paper by Orlhac, et al [3, 4]. These six TFs, entropy, homogeneity, LGZE, HGZE, and short- and long-run emphasis, were reported to be the most robust with respect to tumor region delineation and relatively independent from one another. Homogeneous lesions are known to have higher values of homogeneity, LGZE, and long-run emphasis and lower values of entropy, HGZE, and short-run emphasis than visually heterogeneous lesions [4]. All TFs used in this study are listed in the Supplemental File. Volume of interests with a threshold of 40% SUVmax were placed semi-automatically on target lesions. Volume of interests were set not to include physiological FDG uptake. To extract the TFs, we equalized the histograms by rescaling the intensity with 64 gray levels between the absolute minimum and maximum values in each volume of interest. % change in each TF was defined as [(metrics in DDG/metrics in STD - 1) × 100] (%).

Quantitative analyses were also performed in the group based on either lesion location, LD, SUVmax, or MTV. High- and low LD, high- and low SUVmax, and high- and low MTV groups were defined by the classification using the median of LD, SUVmax, and MTV, respectively.

### Statistical analysis

Visual scores, SUVmax, MTV, and TFs were compared between STD and DDG images with a Wilcoxon signed-rank test, as visual scores were ordinal variables, and SUVmax, MTV, and TFs showed non-normal distributions. % change in SUVmax and % change in MTV were compared among the groups with a *t* test or a Tukey’s HSD test. Spearman’s rank correlation test was used to assess the correlation between LD, SUVmax, and MTV in STD and % change in SUVmax or % change in MTV.

TFs were also assessed as to stability using a Lin’s concordance correlation coefficient (ρc) and % change obtained from STD and DDG images according to previous reports [5, 6]. TFs with high- and low stability were defined as those with (ρc > 0.9 and % change in TF < 5%) and (ρc < 0.8 and % change in TF > 10%), respectively.

All data were statistically analyzed using JMP Pro software (ver17.1.0, SAS Institute Inc., Cary, USA), and a p value less than 0.05 was considered statistically significant. Data are presented as median (interquartile range: IQR) or mean ± standard deviation, depending on the distribution of values unless otherwise indicated.

## Results

### Visual analysis

The mean and median (IQR) scores for lesion blurring were 1.2 and 1 (1–2) for STD and 1.6 and 2 (1–2) for DDG PET images, respectively. The mean and median (IQR) scores for lesion conspicuity were 1.7 and 2 (1–2) in STD and 1.9 and 2 (2–2) in DDG images, respectively. The scores for lesion blurring and conspicuity in DDG were both significantly higher than those in STD images (*p* <0.0001 for both). Increases in the scores of lesion blurring and conspicuity were observed in 34% and 24% of all lesions, respectively. Details of the scores for lesion blurring and conspicuity in STD and DDG PET images are listed in Table 2.

**Table 2:** Scores of lesion blurring and conspicuity in STD and DDG PET images

Score in STD	Score in DDG	Blurring (numbers)	Conspicuity (numbers)
	0	0	0
0	1	10	10
	2	0	3
	0	0	0
1	1	84	5
	2	66	40
	0	0	0
2	1	0	0
	2	63	165

*STD* standard, *DDG* data-driven gating

**Fig. 2:**
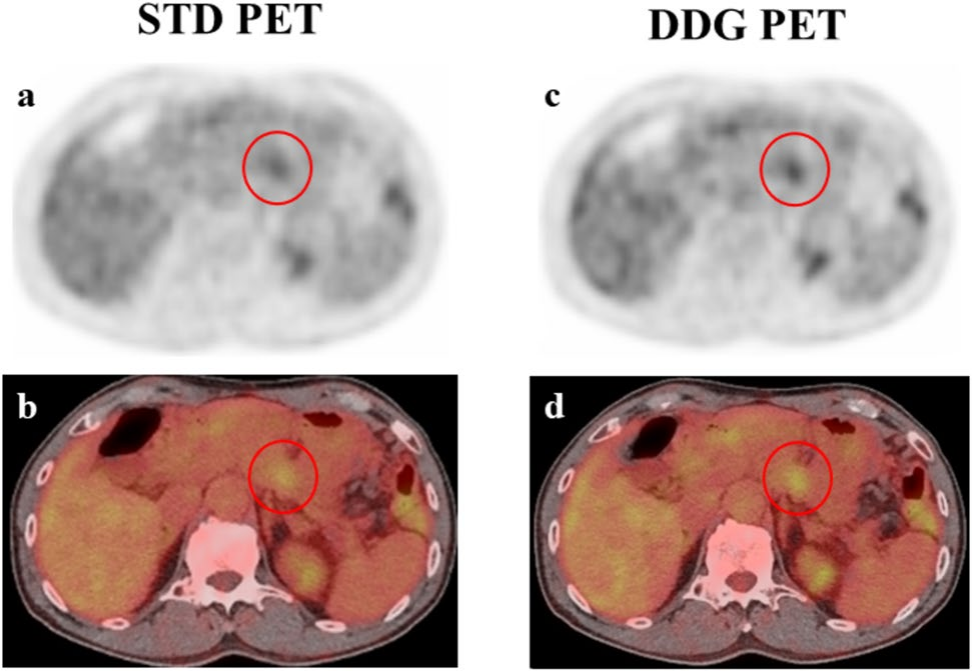
Pancreatic cancer. The pancreatic cancer lesion was visualized with less blurring and more clearly in DDG than in STD FDG PET. The scores of lesion blurring and conspicuity were 0 and 0 in STD and 1 and 1 in DDG PET images, respectively. *STD*: standard free-breathing, *DDG*: data-driven respiratory gating. **a**, **c**: Transaxial FDG PET image, **b**, **d**: fused FDG PET/CT image

Figure 2 shows a representative case with increases in the scores of lesion blurring and conspicuity in DDG PET.

### Quantitative analysis

Details of SUVmax and MTV in STD and DDG images are shown in Table 3.

**Table 3:** SUVmax and MTV in STD and DDG PET images

Location		SUVmax			MTV (ml)		
		STD	DDG	% change	STD	DDG	% change
All		5.3 (3.5–8.3)	6.6 (4.1–10.3)*	21.2 ± 15.4	4.2 (1.8–12.0)	3.2 (1.4–8.6)*	−21.0 ± 27.6
	Breast	5.0 (3.0–8.2)	6.2 (3.7–9.2)*	19.4 ± 13.2	3.1 (1.5–8.6)	2.4 (1.0–7.2)*	−22.7 ± 20.5
Upper abdomen		6.1 (4.7–8.5)	7.8 (5.3–11.4)*	24.3 ± 18.2^†^	6.0 (2.6–14.5)	4.3 (2.2–10.3)*	−18.3 ± 36.2
	Liver	6.7 (4.9–9.2)	8.9 (5.8–12.2)*	25.5 ± 18.4^†^	6.6 (2.5–19.5)	4.3 (2.2–14.4)*	−20.7 ± 36.1
	Pancreas	4.0 (3.4–6.7)	4.4 (3.9–9.5)*	19.7 ± 16.9	4.8 (3.1–8.4)	4.1 (2.1–7.1)**	−9.1 ± 36.3

Expressed as median (interquartile range) or mean ± standard deviation

STD: standard, DDG: data-driven gating

^*^p < 0.0001 or **p < 0.05 vs. values in STD images

^†^p < 0.05 vs. values in breast group

**Fig. 3:**
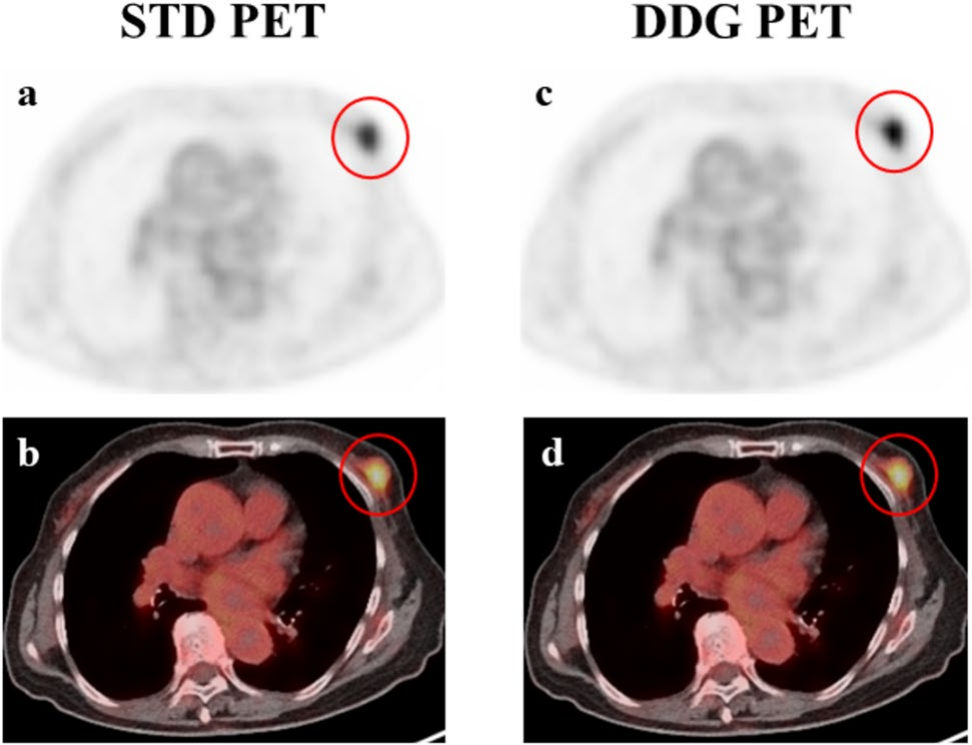
Left breast cancer. The left breast cancer lesion was visualized with less blurring in DDG than in STD FDG PET. SUVmax and MTV in DDG (5.9 and 2.9 ml) were higher and lower, respectively, than those (4.9 and 3.9 ml) in STD PET. *STD*: standard free-breathing, *DDG*: data-driven respiratory gating. **a**, **c**: Transaxial FDG PET image, **b**, **d**: fused FDG PET/CT image

**Fig. 4:**
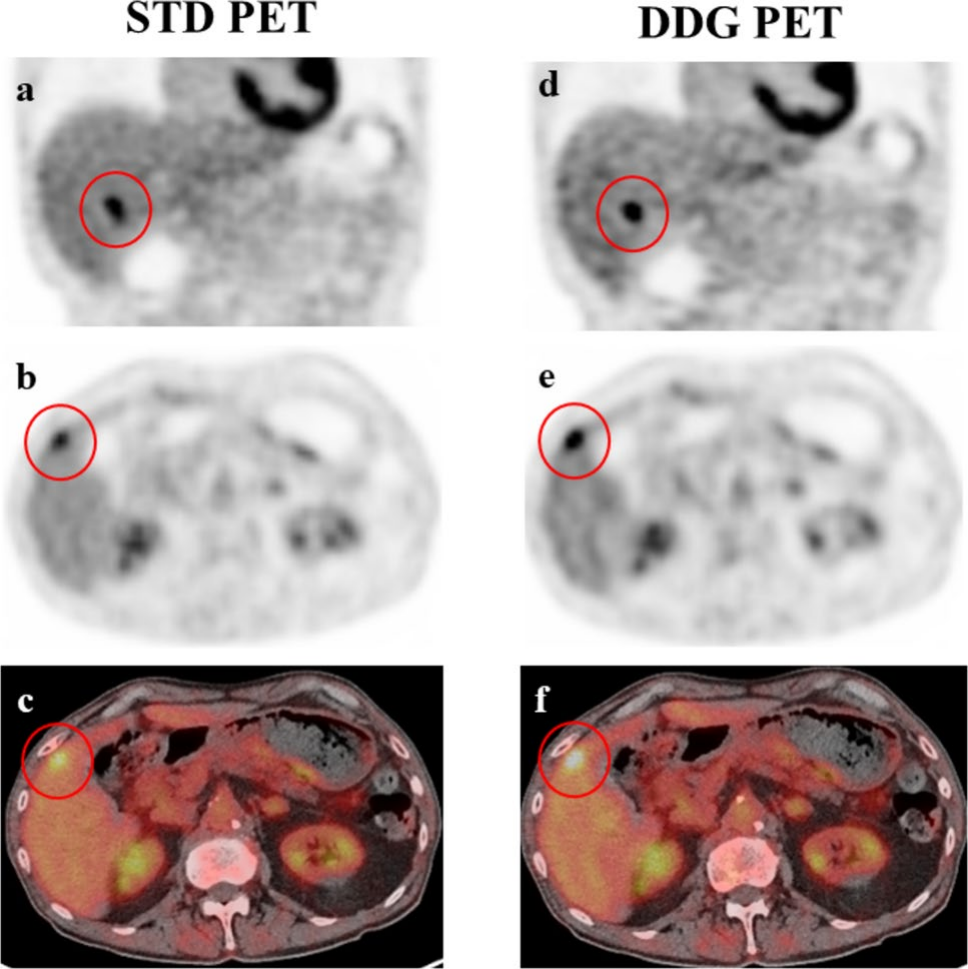
Liver cancer. The liver cancer lesion was visualized with less blurring in DDG than in STD FDG PET. SUVmax and MTV in DDG (9.2 and 2.3 ml) were higher and lower, respectively, than those (5.7 and 5.9 ml) in STD PET. *STD*: standard free-breathing, *DDG*: data-driven respiratory gating. **a**, **d**: Coronal FDG PET image, **b**, **e**: transaxial FDG PET image, **c**, **f**: fused FDG PET/CT image

In all lesions, SUVmax and MTV were 5.3 (3.5–8.3) and 4.2 (1.8–12.0) ml in STD and 6.6 (4.1–10.3) and 3.2 (1.4–8.6) ml in DDG images, respectively. SUVmax and MTV in DDG were significantly higher and lower, respectively, than those in STD images (*p* < 0.0001 for both). There was a strong correlation between SUVmax in STD and DDG images (*ρ* = 0.97, *p* < 0.0001) and between MTV in STD and DDG images (*ρ* = 0.95, *p* < 0.0001). Figures 3 and 4 show cases with an increase in SUVmax and a decrease in MTV in DDG PET.

The % change in SUVmax and % change in MTV were 21.2 ± 15.4% and −21.0 ± 27.6%, respectively. An increase in SUVmax and a decrease in MTV were observed in 96% and 86% of all lesions, respectively. No correlations were observed between % increase in SUVmax or % increase in MTV and LD, SUVmax, or MTV in STD images.

#### Quantitative analysis in the groups by lesion location

In both the breast and upper abdominal groups, SUVmax and MTV in DDG were significantly higher and lower, respectively, than those in STD images (*p* <0.0001 for all, Table 3). % change in SUVmax was significantly greater in the upper abdominal group than in the breast group (24.3 ± 18.2% vs. 19.4 ± 13.2%, *p* < 0.05). No significant difference in % change in MTV was observed between the groups. No correlations were observed between % increase in SUVmax and % increase in MTV and LD, SUVmax, and MTV in STD images in the breast group. However, % increase in MTV was negatively correlated with LD or MTV in STD (*ρ*= −0.42, −0.45, respectively, *p* <0.005 for both) in the upper abdominal group.

The upper abdominal group was further divided into the liver and pancreas subgroups to analyze the data in greater detail. In both the liver and pancreas groups, SUVmax and MTV in DDG were significantly higher and lower, respectively, than those in STD images (*p* < 0.05 for MTV in pancreas group, *p* < 0.0001 for others, Table 3). % change in SUVmax was significantly higher in the liver group than in the breast group (25.5 ± 18.4% vs. 19.4 ± 13.2%, *p* < 0.05). No significant difference was observed in % change in MTV among the three groups.

#### Quantitative analysis in the high and low groups

% change in SUVmax or MTV was compared between the high and low groups classified according to median LD, SUVmax, or MTV in STD PET images (Table 4). In the high LD and high MTV groups, % change in MTV was significantly greater than in the low LD and low MTV groups, respectively (*p* < 0.05 for LD, *p* < 0.005 for MTV). No significant differences in % change in MTV were observed between high- and low SUVmax groups. With regard to % change in SUVmax, no significant differences were observed between the high and low groups for LD, SUVmax, and MTV.

**Table 4:** % change in SUVmax and MTV in high and low groups

Location		Factors for classification	% change in SUVmax		% change in MTV	
			High gr	Low gr	High gr	Low gr
All		LD	21.4 ± 17.3	21.1 ± 13.7	−26.0 ± 24.2	−16.9 ± 29.5*
		SUVmax	22.1 ± 18.0	20.4 ± 12.4	−22.5 ± 23.4	−19.6 ± 31.2
		MTV	21.6 ± 14.0	20.9 ± 16.8	−26.3 ± 21.2	−15.8 ± 32.0**
	Breast	LD	16.3 ± 13.8	21.9 ± 12.2*	−19.8 ± 15.3	−25.1 ± 23.7
		SUVmax	17.1 ± 14.4	21.6 ± 11.5*	−19.5 ± 14.8	−26.0 ± 24.6
		MTV	17.3 ± 13.8	21.4 ± 12.2	−21.0 ± 17.3	−24.5 ± 23.2
	Upper abdomen	LD	25.1 ± 16.1	23.5 ± 20.3	−33.8 ± 20.9	−2.3 ± 41.6^†^
		SUVmax	30.2 ± 19.8	18.5 ± 14.4**	−28.9 ± 27.4	−7.9 ± 40.9^††^
		MTV	25.6 ± 16.0	23.0 ± 20.2	−33.6 ± 20.3	−3.3 ± 42.0^†^

*gr* group, *LD* longest diameter

^*^p < 0.05, **p < 0.005, ^†^p < 0.0001, or ^††^p < 0.01 vs. values in high group

These analyses were also performed in the breast and upper abdominal groups (Table 4). In the breast group, a significantly greater % change in SUVmax was observed in the low LD and low SUVmax groups than in the high LD and high SUVmax groups, respectively (*p* < 0.05 for both). In the upper abdominal group, % change in SUVmax was significantly greater in the high than in the low SUVmax groups (*p* < 0.005) and % change in MTV was significantly greater in the high than in the low groups regarding LD, SUVmax, and MTV (*p*< 0.0001 for LD and MTV, *p* < 0.01 for SUVmax).

#### Texture analysis

Lin’s concordance correlation coefficient (ρc), % change in TFs, and statistical differences between STD and DDG PET images are listed in the Supplemental Table.

Based on the definition using a Lin’s concordance correlation coefficient and % change obtained from STD and DDG images, 5 (9%) and 15 (27%) of 56 TFs were classified as TFs with high- and low stability, respectively. Statistical differences were observed in 41 (73%) of 56 TFs between STD and DDG images, which included entropy, homogeneity, LGZE, and long-run emphasis of the 6 recommended TFs as mentioned above. Changes in entropy and LGZE indicated that lesions in DDG exhibited more homogeneous FDG uptake than those in STD, whereas changes in homogeneity and long-run emphasis indicated that lesions in DDG exhibited more heterogeneous FDG uptake than those in STD images.

In the breast group, 5 and 15 TFs were classified as TFs with high- and low stability, respectively. Thirty-one TFs showed statistical differences between STD and DDG. Similarly, in the upper abdominal group, 4 and 17 TFs were classified as high- and low stability, respectively. Thirty-five TFs showed statistical differences between STD and DDG.

## Discussion

In this study, we evaluated the effect of DDG on the visualization and quantification of breast and upper abdominal cancers in FDG PET/CT. The DDG system used in this study does not require any external equipment or extended examination time and is expected to become widely available in the near future. Since many of body tumors are affected by respiratory motion in imaging, data on malignancies other than lung cancer are also considered valuable.

To the best of our knowledge, this is the first study to investigate the effects of DDG with a special focus on breast and upper abdominal cancers. DDG was demonstrated to improve image quality and lesion depiction by reducing the motion blur caused by respiratory motion. The results of the visual assessment were consistent with those of previous studies, mainly in lung cancer [2, 7]. The results of this study should facilitate the further use of DDG in clinical PET imaging.

This study also demonstrated that SUVmax increased and MTV decreased with DDG in all, breast, or upper abdominal lesions. These results are also consistent with previous findings mainly in lung cancer [2, 7–9]. The decrease in MTV is attributed to the precise delineation of tumor boundaries by DDG. % change in SUVmax was significantly greater in the upper abdominal group than in the breast group. This result was likely due to the location of the upper abdominal lesions, which are close to the diaphragm and highly affected by respiration. Kang, et al. reported similar observations in a comparison of lesions in the upper and lower lung lesions [8]. Although these changes in quantitative metrics along with improvements in lesion depiction may not directly lead to diagnostic results, DDG is expected to have an impact on the diagnostic process due to clearer PET images.

The effect of respiratory correction is known to be related to lesion size or volume as well as lesion location [10]. Quantitative analysis in high and low groups in all lesions demonstrated that % changes in MTV were greater in the high LD and high MTV groups in this study. These results appeared different from previous reports in which the effect of respiratory correction or gating was observed more frequently in small lesions [10, 11]. However, % change in SUVmax was greater in the low LD group when only breast cancer was analyzed in this study. In the upper abdominal group, a significantly greater % change in MTV was observed in the high LD and high MTV groups. It is possible that the effect in the upper abdominal lesions was more strongly reflected in the analysis of all lesions, which may have resulted in a reduction in the effect on % change in small lesions. The validity of the greater effect on changes in MTV in larger lesions should be determined in accordance with future studies.

Regarding the changes in TFs with DDG, high stability was observed in a limited number of features in this study, which was consistent with a report dealing with a large number of TFs [12]. Another clinical study reported the low stability of TFs using respiratory gating [13], while other reports reported high stability [5, 6]. The types of malignancies and/or the locations or sizes of the lesions evaluated could be possible reasons for this discrepancy. It should be noted that this study was conducted in a relatively large number of patients with breast and upper abdominal cancers, whereas previous studies reporting high stability were conducted in a small number of patients solely with lung cancer [5, 6]. Changes in LGZE with DDG were observed either in all, breast or upper abdominal lesions in this study. This finding was also consistent with a previous report that TFs based on gray-level size zone matrix, in which LGZE was included, were significantly affected by respiratory motion [14].

This study has some limitations. First, this study was not conducted using a digital PET/CT scanner. As a growing number of PET/CT scanners are being converted to digital PET/CT scanners, it remains to be seen whether the same results can be obtained with DDG in digital PET/CT scanners, as observed in the present study. Second, this study focused only on the comparison of visual and quantitative results between STD and DDG PET images in the lesions visualized on STD images. The effect of DDG on lesion detection was not evaluated. An increase in SUVmax may have resulted in new true- or false-positive lesions visualized only on DDG PET images. However, DDG images are currently considered to be used to confirm the findings of STD images in clinical practice because DDG images are easily obtained without additional data acquisition time. Unless there is a significant change in the comparison between the two, it is unlikely that DDG will change the diagnosis. Further studies are warranted to assess the impact of additional information from DDG on diagnosis and the diagnostic accuracy of DDG images alone.

In conclusion, this study demonstrated that device-less DDG improved visualization and quantification of breast and upper abdominal cancers in FDG PET/CT without increasing examination time. DDG PET image exhibited an increase in SUVmax, a decrease in MTV, and changes in TFs.

## Supplementary Information

Below is the link to the electronic supplementary material.

Supplementary file1 (DOCX 41 kb)

## Data Availability

The datasets generated and analyzed during the current study are available from the corresponding authur on reasonable request.
