# Supplementary material for: Effects of data-driven respiratory gating on visualization and quantification of breast and upper abdominal cancers in FDG PET/CT examinations
Source: Ann Nucl Med. 2025 Jan 23;39(5):450–7. doi: 10.1007/s12149-025-02017-8 (PMC12014764; doi:10.1007/s12149-025-02017-8)
Supplement: Supplementary file 1 — Supplementary file1 (DOCX 41 kb) [file 12149_2025_2017_MOESM1_ESM.docx]

**Supplemental Table**

**Texture features used in this study**

| SUV Histogram | Variance |  | NGLCM3D | Contrast max |
| --- | --- | --- | --- | --- |
|  | Entropy |  |  | Homogeneity mean |
| NGLCM | Uniformity mean |  |  | Homogeneity max |
|  | Uniformity max |  |  | Correlation mean |
|  | Entropy mean |  |  | Correlation max |
|  | Entropy max |  | NGLCM3DMean | Uniformity |
|  | Dissimilarity mean |  |  | Entropy |
|  | Dissimilarity max |  |  | Dissimilarity |
|  | Contrast mean |  |  | Contrast |
|  | Contrast max |  |  | Homogeneity |
|  | Homogeneity mean |  |  | Correlation |
|  | Homogeneity max |  | GLSZM | High Gray Level Zone Emphasis |
|  | Inverse Difference Moment mean |  |  | Low Gray Level Zone Emphasis |
|  | Inverse Difference Moment max |  |  | Large Area Emphasis |
|  | Correlation mean |  |  | Small Area Emphasis |
|  | Correlation max |  |  | Intensity Variability |
| NGTDM | Coarseness |  |  | Run Length Variability |
|  | Contrast |  |  | Zone Percentage |
|  | Busyness |  |  | Short Runs Emphasis mean |
|  | Complexity |  |  | Short Runs Emphasis max |
|  | Strength |  |  | Long Runs Emphasis mean |
| NGLCM3D | Uniformity mean |  |  | Long Runs Emphasis max |
|  | Uniformity max |  |  | Gray Level Nonuniformity mean |
|  | Entropy mean |  |  | Gray Level Nonuniformity max |
|  | Entropy max |  |  | Run Length Nonuniformity mean |
|  | Dissimilarity mean |  | GLRLM | Run Length Nonuniformity max |
|  | Dissimilarity max |  |  | Run Percentage mean |
|  | Contrast mean |  |  | Run Percentage max |

NGLCM: Normalized Gray Level Cooccurrence Matrix, NGTDM: Neighborhood Gray Tone Difference Matrix,

NGLCM3D: 3D Normalized Gray Level Cooccurrence Matrix, GLSZM: Gray Level Size Zone Matrix, GLRLM: Gray Level Run Length Matrix

**Lin’s concordance correlation coefficient (ρc), % change in texture features, and statistical difference between STD and DDG PET images**

**in all, breast, and upper abdominal cancer lesions**

|  |  | All | | |  | Breast | | |  | Upper abdomen | | |
| --- | --- | --- | --- | --- | --- | --- | --- | --- | --- | --- | --- | --- |
|  |  | ρc | % change | p value |  | ρc | % change | p value |  | ρc | % change | p value |
| SUV Histogram | Variance | 0.807671 | 4.171255 | 0.0158 |  | 0.843541 | 4.065295 | 0.0186 |  | 0.675792 | 3.253726 | 0.3675 |
|  | Entropy | 0.863592 | -2.63329 | <.0001 |  | 0.869691 | -3.60963 | <.0001 |  | 0.823019 | -3.29321 | <.0001 |
| NGLCM | Uniformity mean | 0.735436 | 56.39535 | <.0001 |  | 0.669488 | 29.65009 | <.0001 |  | 0.917452 | 47.82609 | 0.0003 |
|  | Uniformity max | 0.600682 | 61.5 | <.0001 |  | 0.538008 | 46.15385 | <.0001 |  | 0.898372 | 46.56489 | 0.0009 |
|  | Entropy mean | 0.924599 | -8.99102 | <.0001 |  | 0.936602 | -7.35632 | <.0001 |  | 0.885184 | -8.61988 | <.0001 |
|  | Entropy max | 0.931634 | -7.90744 | <.0001 |  | 0.946404 | -6.62864 | <.0001 |  | 0.891526 | -8.70765 | <.0001 |
|  | Dissimilarity mean | 0.87595 | 5.014234 | <.0001 |  | 0.889186 | 5.063924 | <.0001 |  | 0.800452 | 8.300791 | <.0001 |
|  | Dissimilarity max | 0.837607 | 7.904684 | <.0001 |  | 0.82954 | 6.358658 | <.0001 |  | 0.809267 | 6.565567 | 0.0039 |
|  | Contrast mean | 0.845854 | 13.21751 | <.0001 |  | 0.856249 | 10.62246 | <.0001 |  | 0.737861 | 20.79759 | <.0001 |
|  | Contrast max | 0.769374 | 15.89391 | <.0001 |  | 0.756898 | 8.883028 | <.0001 |  | 0.733515 | 16.69816 | 0.0035 |
|  | Homogeneity mean | 0.771417 | -2.98954 | 0.0001 |  | 0.709536 | -0.82775 | 0.0389 |  | 0.830929 | -4.59219 | 0.0004 |
|  | Homogeneity max | 0.331252 | -3.34467 | 0.0927 |  | 0.25722 | 1.646214 | 0.8974 |  | 0.621372 | -7.93737 | 0.0019 |
|  | Inverse Difference Moment mean | 0.636824 | -3.62319 | 0.0201 |  | 0.507336 | -2.35383 | 0.293 |  | 0.782318 | -8.27858 | 0.0135 |
|  | Inverse Difference Moment max | 0.250633 | -4.9812 | 0.2946 |  | 0.179218 | 1.210287 | 0.9427 |  | 0.582883 | -5.27728 | 0.0503 |
|  | Correlation mean | 0.628699 | -18.1818 | 0.0158 |  | 0.675653 | -29.4118 | <.0001 |  | 0.478115 | 3.833866 | 0.3174 |
|  | Correlation max | 0.57482 | -10.2253 | 0.5243 |  | 0.607267 | -19.9603 | 0.0024 |  | 0.493718 | 25.25849 | 0.0151 |
| NGTDM | Coarseness | 0.755505 | 28.97959 | <.0001 |  | 0.756008 | 3.28E+15 | <.0001 |  | 0.636752 | 3.301887 | 0.0055 |
|  | Contrast | 0.219476 | -34.5455 | <.0001 |  | 0.191703 | -100 | 0.0101 |  | 0.230278 | 96.61836 | <.0001 |
|  | Busyness | 0.064348 | -18.9751 | 0.6734 |  | 0.042072 | -100 | 0.3516 |  | 0.293033 | 4.404404 | 0.6133 |
|  | Complexity | 0.620087 | -41.1683 | 0.0045 |  | 0.675266 | -100 | 0.635 |  | 0.475585 | 50.16041 | 0.0007 |
|  | Strength | 0.661486 | -28.4904 | 0.003 |  | 0.684328 | -100 | 0.0831 |  | 0.594345 | 4.319531 | 0.0266 |
| NGLCM3D | Uniformity mean | 0.715623 | 51.07527 | <.0001 |  | 0.66953 | 37.14286 | <.0001 |  | 0.840589 | 50.83333 | 0.0003 |
|  | Uniformity max | 0.661372 | 59.91379 | <.0001 |  | 0.637777 | 69.13747 | <.0001 |  | 0.714251 | 70.62937 | 0.0015 |
|  | Entropy mean | 0.924255 | -9.31173 | <.0001 |  | 0.938453 | -8.49765 | <.0001 |  | 0.881358 | -8.98719 | <.0001 |
|  | Entropy max | 0.933694 | -7.40053 | <.0001 |  | 0.951512 | -6.01816 | <.0001 |  | 0.888419 | -7.83672 | <.0001 |
|  | Dissimilarity mean | 0.878564 | 5.203407 | <.0001 |  | 0.916132 | 2.937803 | <.0001 |  | 0.773988 | 10.78074 | <.0001 |
|  | Dissimilarity max | 0.82303 | 7.658446 | <.0001 |  | 0.821477 | 5.352945 | <.0001 |  | 0.787999 | 7.467256 | <.0001 |
|  | Contrast mean | 0.86462 | 11.14361 | <.0001 |  | 0.897489 | 7.39125 | <.0001 |  | 0.737971 | 14.98973 | <.0001 |
|  | Contrast max | 0.758942 | 13.46489 | <.0001 |  | 0.741037 | 11.45171 | <.0001 |  | 0.762861 | 27.53524 | <.0001 |
|  | Homogeneity mean | 0.804014 | -3.25139 | <.0001 |  | 0.794539 | 1.678556 | 0.1058 |  | 0.80342 | -9.03747 | <.0001 |
|  | Homogeneity max | 0.203334 | 0 | 0.9304 |  | 0.165505 | 3.017241 | 0.1074 |  | 0.492618 | -2.41978 | 0.0354 |
|  | Correlation mean | 0.614261 | -30.3704 | 0.0095 |  | 0.682996 | -51.6854 | 0.0007 |  | 0.444114 | -22.5681 | 0.7777 |
|  | Correlation max | 0.601106 | -1.77112 | 0.6695 |  | 0.640669 | -8.57558 | 0.1625 |  | 0.542819 | 16.44172 | 0.023 |
| NGLCM3DMean | Uniformity | 0.669153 | 1.863354 | <.0001 |  | 0.641102 | -16.8831 | <.0001 |  | 0.733586 | 50 | 0.0007 |
|  | Entropy | 0.889416 | -8.42374 | <.0001 |  | 0.911013 | -13.8904 | <.0001 |  | 0.819942 | -7.67437 | <.0001 |
|  | Dissimilarity | 0.585124 | 1.195393 | 0.0002 |  | 0.540233 | -3.83015 | 0.0001 |  | 0.718369 | 14.15325 | 0.1971 |
|  | Contrast | 0.607172 | 7.819902 | <.0001 |  | 0.579969 | -5.39437 | <.0001 |  | 0.651449 | 39.84248 | 0.0185 |
|  | Homogeneity | 0.78067 | -4.84183 | 0.0018 |  | 0.786583 | -14.5381 | <.0001 |  | 0.731397 | 0.807382 | 0.7168 |
|  | Correlation | 0.546538 | -7.00483 | 0.3712 |  | 0.709095 | -8.70787 | 0.013 |  | 0.290937 | -11.0535 | 0.2936 |
| GLSZM | High Gray Level Zone Emphasis | 0.688044 | -5.41625 | 0.1656 |  | 0.790881 | 0.272676 | 0.9377 |  | 0.562623 | -10.8524 | 0.024 |
|  | Low Gray Level Zone Emphasis | 0.449396 | 13.54167 | <.0001 |  | 0.376641 | 19.37603 | 0.0006 |  | 0.531106 | 11.95446 | 0.0002 |
|  | Large Area Emphasis | 0.619998 | -0.5619 | 0.0218 |  | 0.476661 | -0.32015 | 0.1484 |  | 0.727986 | -2 | 0.0576 |
|  | Small Area Emphasis | 0.512906 | 0.601738 | 0.0345 |  | 0.383586 | 0.30273 | 0.1454 |  | 0.64598 | 1.18155 | 0.1034 |
|  | Intensity Variability | 0.936269 | 0.803213 | 0.5042 |  | 0.906549 | -2.36356 | 0.9033 |  | 0.948055 | 1.591015 | 0.384 |
|  | Run Length Variability | 0.947207 | -1.17208 | 0.6035 |  | 0.9325 | -4.57058 | 0.9587 |  | 0.954894 | 0.123793 | 0.4108 |
|  | Zone Percentage | 0.579543 | 0.560601 | 0.0362 |  | 0.463889 | 0.332668 | 0.1804 |  | 0.688266 | 2.035419 | 0.0758 |
|  | Short Runs Emphasis mean | 0.515611 | 0.070807 | 0.0187 |  | 0.36689 | 0.060634 | 0.0778 |  | 0.672377 | 0.11146 | 0.1015 |
|  | Short Runs Emphasis max | 0.72865 | 0 | 0.0005 |  | 0.669323 | 0 | 0.0774 |  | 0.758039 | 0.502513 | 0.0013 |
|  | Long Runs Emphasis mean | 0.5531 | -0.37256 | 0.029 |  | 0.40116 | -0.14876 | 0.117 |  | 0.699395 | -0.41809 | 0.1173 |
|  | Long Runs Emphasis max | 0.167505 | -0.31127 | 0.1157 |  | 0.107066 | -0.39897 | 0.2963 |  | 0.379566 | -0.10049 | 0.2425 |
|  | Gray Level Nonuniformity mean | 0.945297 | 0.914381 | 0.6478 |  | 0.919759 | -1.48883 | 0.9697 |  | 0.952251 | 16.92661 | 0.4348 |
|  | Gray Level Nonuniformity max | 0.942761 | 3.504482 | 0.6744 |  | 0.921889 | -1.52519 | 0.9579 |  | 0.948451 | 18.31369 | 0.4622 |
|  | Run Length Nonuniformity mean | 0.961315 | -4.62882 | 0.8129 |  | 0.942997 | -12.5578 | 0.8441 |  | 0.966461 | -4.76283 | 0.5809 |
| GLRLM | Run Length Nonuniformity max | 0.959411 | -7.90506 | 0.912 |  | 0.948444 | -11.94 | 0.8178 |  | 0.962398 | -1.00853 | 0.6718 |
|  | Run Percentage mean | 0.604101 | 0.154289 | 0.0264 |  | 0.485842 | 0.087211 | 0.1076 |  | 0.703594 | 0.279185 | 0.1097 |
|  | Run Percentage max | 0.734017 | 0 | 0.0011 |  | 0.673864 | 0 | 0.0858 |  | 0.766206 | 1.317123 | 0.003 |

STD: standard free-breathing, DDG: data-driven respiratory gating, NGLCM: Normalized Gray Level Cooccurrence Matrix, NGTDM: Neighborhood Gray Tone Difference Matrix, NGLCM3D: 3D Normalized Gray Level Cooccurrence Matrix, GLSZM: Gray Level Size Zone Matrix, GLRLM: Gray Level Run Length Matrix
